# Making Money

**Published:** 1863-08

**Authors:** 


### MAKING MONEY

Is about one of the best promoters of health we at present think of; it enlivens the mind, which induces greater bodily activities, and these, as all know, are better than any medicine for the securement of health and the prevention of disease. The same amount of muscular effort, expended in an encouragingly remunerative employment, will do many times more good toward removing and preventing sickness, than if it was merely and solely intended for that end. But it takes more of a man, requires more mind, more moral force, to save money than to make it; the idea being expressed, although in not very elegant phrase, "Any fool or knave can make money, but it requires a wise man to keep it," to save it. In fact, poor Old Richard used to say: "A penny saved is tuppence gained." A man is a man, in proportion to the amount of self-denial he can exercise over himself, in proportion to his moral courage to deny himself as to his appetites and gratifications. Spendthrifts have none of these high qualities, consequently, the world over, they are contemptuously set aside with the expression: "Oh! he's no account." Where there is most poverty, there is most crime, destitution, disease, and premature death; so that it is not by any means out of place in a journal of health, to suggest what may promote the comfort of its readers and show how they can economize; and when that economy may be practiced without involving self-denial, but actually saves time, trouble, and labor, it is a thing worthy of consideration. Hence, with great confidence, we direct attention to the following article on

### HEALTH, COMFORT, AND ECONOMY,

Which are all largely promoted by the use of an apparatus patented within the present year by Warren L. Fish, for boiling, frying, stewing, and steeping food, and toasting bread, with the flame that lights the room, at an expense of gas (at twenty-five cents per hundred feet) of less than two cents an hour; if kerosene oil is used, the cost is still less. There is no dust, dirt, or ashes, and the varying circumstances to which the apparatus is applicable, would be scarcely conjectured at first sight; it is handy for young and old, for sick and well, for the laborer and the student, night or day, summer or winter. A single match will put it in operation instantly, and in fifteen minutes, cold water is made to boil. Coal is selling, at this writing, at ten dollars a ton in New-York, while at Boston and Cincinnati it is twelve dollars. At these prices a breakfast which it would cost twelve cents to cook in a common range, which is a city cooking-stove, would cost one cent; can be prepared by Fish's lamp at a less cost than the price of the wood necessary to kindle the coal in the range, to say nothing of the trouble of kindling and subsequent cleaning up of ashes, cinders, and the dust over the whole room. Kindling a fire in a stove or range in midsummer, adds greatly to the heat of the whole house, even when the range is in the basement or cellar.

In summer-time, none but actual laborers ought to take any thing for breakfast and supper but cold or toasted bread and butter and a cup or two of hot tea or coffee, which can be prepared at the cost of a cent or two for a whole family. By having one of these lamps, a clerk or student could save ten times or more the price of the lamp in a year, by simply preparing his own breakfast, while persons who board, or go to the country for the summer, would by its use be able to add greatly to their comfort by preparing their own tea or coffee, and little delicacies in case of sickness. But as an article for the sick-room and the nursery, this lamp is invaluable; no mother of a growing family, if she knew its value, would be willing to do without it for a day. The husband who has any manly feeling for the wife of his bosom, and even a slight concern for the lives of his young children, would sooner live on bread and water for a month, if necessary, to save the two dollars requisite to purchase one of these admirable household articles, than to be without one of them for a week, simply because any child is liable to be taken sick any night, and in almost all the illnesses of childhood, warm water is more or less necessary; it is a prompt and perfect cure for

Croup,

One of the most dangerous of all the diseases of young children, often destroying life in a few hours, simply by having boiling water, with two or three cloths in it, to be applied alternately to the throat, as hot as can be borne with the hand, the feet and hands to be kept warm all the time, the whole body also being wrapped up warm, and the water not being allowed to dribble on the clothing; this to be continued until the cough is loosened and the little patient breathes easy and falls asleep. By one of these lamps, water can be made to boil in ten minutes, or even less, while if a fire has to be kindled in a range or stove, or ordinary fire-place, a very much longer time would be necessary. But as a convenience for the sick-room in general, Fish's Lamp is literally invaluable. For hospitals, for the tent or barrack of the stationed soldier, for camping out, for picnics, going a-fishing, etc., it is an article of comfort beyond all proportion to its cost. Circulars will be given on application, or sent, free of charge, by addressing Wm. D. Russell, Agent, 206 Pearl street, New-York.

Various articles have been introduced in these pages to the notice of our subscribers, but not until we have seen for ourselves that it was all that was claimed for them, knowing that inventors, patentees, etc., are nearly always too enthusiastic to be truthful, and we were unwilling to be instrumental in palming an imposition on our readers. When requested to notice an article, we always require to be well paid; but we have had occasion to notice books and other things voluntarily, without knowing the proprietors or receiving any compensation for the same before or since, from the sole wish of conferring on our readers a direct pecuniary benefit, and adding to their personal comfort, for between an editor and his habitual readers there grows up a feeling of personal acquaintance and even friendship, without ever having seen each other, and with this there is an instinctive desire for the performance of mutual kind offices. We are not aware that any person who has purchased an article on the faith of our special recommendation of the same, has ever been disappointed; while time, as to ourselves, has but increased the appreciation had, for example, for Worcester's celebrated Hinged Plate Piano; Andrews & Dixon's inimitable low-down grate for open fire-places, burning any kind of coal or wood. Pyle's O. K. Soap we believe the best ever made. Milk sold by the Rockland County and New-Jersey Milk Association, at 146 East Tenth street, near Broadway, drawn daily from farm-house cows, rich, fresh, and unadulterated, all of which the Company guarantees. But now in this number we have four new things to commend, three of which will certainly save time and toil and money; the fourth will add more to the pure satisfaction and pleasurable feelings of our better nature, than can be obtained at as little cost in that direction, in the wide world besides. Fish's Patent Lamp Heating Apparatus has already been named; its conveniences and advantages have been understated rather than exaggerated. Another labor and money-saving patent is a machine for wringing out clothes after having been washed, or rather it removes the water from the fabrics without the straining which attends the ordinary wringing with the strong hands of stout Irish washerwomen. Ladies know that the most costly laces are deprived of the water by simple pressure or squeezing in the hand; to wring them would be but to ruin, showing clearly that wringing clothes causes a destructive strain. If, then, fine lace lasts longer by having the water simply pressed out of them, any other woven fabric will last longer if the water is pressed out of it instead of being wrung, as commonly practiced. Hence the testimony of hotel proprietors, gentlemen of intelligent observation, and women who are notable housekeepers, and have used the UNIVERSAL CLOTHES-WRINGER for several years, not only in favor of the less injury done to clothing, but of the great amount of labor saved in this, one of the most laborious parts of the weekly wash. One of the important advantages of this wringer over all others is, it has no metal about it to rust or stain the clothing.

Another of the new inventions is remarkable in several respects. It does not save money directly, but it saves time to many a hard-worked mother, and it

SAVES BLOOD!!

By destroying a thousand lives in fifty-nine seconds and three quarters. Another queer truth about it is, that it is the product of a brain which one would think would be the very last kind of persons in creation to have thoughts in that direction. Imagine Raphael or Michael Angelo, or a Rubens, devising the best ways and means of

KILLING A LOUSE.

And yet here is a man who we believe is the most accomplished miniature-painter this country has ever produced, a man whose gentleness of nature has become a proverb among his friends, and whom to look at one would suppose would not have the heart to tread on a worm or kill a fly, and yet we have known him for several months to turn his back upon his refined and elevating profession, and has spent day and night and dollars in devising a plan for destroying life in so wholesale a manner, that to number it by thousands a day is mere child's work for his discovery. Possibly his mind has taken this direction from the force of the very significant circumstances, that having spent some of the best years of his life in planting and cultivating, in his mountain home in the distant South, one of the most beautiful, productive, and extensive fruit-farms on this continent, and just as he was settling down in his age to drink in its beauties and luxuriate in its luscious products for the remainder of his life, he was hastily driven from it, because he was a "Union man;" and through rain and mud, and inconceivable privation in a winter's journey over mountains, and gorges, and angry floods, with a wife and a large family of children, arrived at last in Cincinnati, with bare life left; thence he came to this city, and went to painting in Broadway, but all the time indulging in and cherishing thoughts of

### Murder! Murder!

Not of his old secesh friends and neighbors in East-Tennessee, but of all the vermin which infest the persons of humanity, such as lice, nits, and bed-bugs. We have not heard him expatiate on its vital properties as against fleas, because they are never there, but always somewhere else than you hoped or expected to find them; so it would do no good to put a thing "there" to destroy one of the hopping fraternity, when it was sure to be at any other place rather than there. But as much as we liked this murdering artist, after an acquaintance, personal and intimate, for more than twenty years, we had not the faith in his invention which he had, and refused to put it in our journal, which he wanted us to do a month ago; but we wanted the proof positive first. We refused all certificates from commissioners, generals, presidents, and all that, because we knew he was such a clever, good-hearted fellow, that any one who knew him as we did, and there were many such in New-York, would certify to any thing he wanted, and would even lend him money, which of course is the crucial test of human friendship. He thought if HALL'S JOURNAL OF HEALTH would only speak a good word for him, that it would be the entering wedge of a fortune to him. But that was all to no purpose. We in effect spoke thus to him: "We are old friends; I like you, and believe any thing you say; I believe in the Sanitary Commission; I believe in General I——, and all the other generals and colonels and captains, down to izzard, and even the other side of '&c;' but I might 'believe a lie,' like that immense host which is said to be 'bound for the land of' —— a certain sable personage whom it is not necessary to name more specifically. I want ocular demonstration." I wanted more. I wanted not merely to know and see for myself that the little twenty-five cent vial of liquid which he handed me would kill every inhabitant, mother, father, and child, of any five heads of the newest comers to the Five Points Mission, in one minute after its thorough application; but I wanted to know, also, whether, if it was so powerful to do all that, it might not be strong enough to kill the child too, after a few applications. So I asked to know its constituents, that I might prepare some myself, and examine into their nature, and the nature of their chemical products, because two substances may be singly harmless, yet united be deadly. Air is simple and pure; oxygen is the very life of all things that breathe, and yet add a portion more of oxygen to a given amount of air, and it becomes aqua-fortis, one of the most corrosive poisons in nature, for if swallowed, it destroys life instantly. But even if the chemical compound was as hurtless as either of its constituents, and was as efficacious as it was claimed to be, yet I had before me some of the tricks of trade daily practiced by men who sell patent medicines, bitters, sarsaparillas, etc., for they will come to a chemist with a genuine article, pay him five, fifty, or five hundred dollars for his analysis and certificate. All that seems plain sailing; but then, there was this drawback: that was the only "true blue" bottle ever manufactured, or intended to be; all the others were to be made of molasses and water, or of alcohol and tanzy, indefinitely diluted with cold water. But said he: "Doctor, you know me, and I know you, but if I tell it to you, it will no longer be a secret; it might leak out, and my fortune would be lost." That was very true, but I solved the knot by assuring him that I would not tell my wife about it. He was at once satisfied, made a clean breast of it, I obtained the materials, mixed them together, and lo! and behold, all was right. But it was not enough. I had neither time nor inclination to be fooling about in making

A LOUSY EXPERIMENT.

I said to him: "Go down to the Five Points. Mr. Barlow has plenty of subjects new and fresh every day; then bring me a letter in the handwriting of that morally brave, that good, energetic, heroic, and self-denying man, and then I will see about it;" and sure enough he brought me yesterday, July 10th, 1863, the following in black and white, dated Five Points House of Industry, New-York, July 10th, 1863:

"FIVE POINTS HOUSE OF INDUSTRY, NEW-YORK, July 10th, 1863.

"J. W. DODGE: DEAR SIR: I have this morning made a special test of your Infallible Vermin Exterminator upon some of the children in this Institution, in order to ascertain how quickly your specific will act in the destruction of the vermin, and find, by personal observation, that in one minute after the fluid is applied, the lice are all dead, and the relief complete. The life of the nits is always destroyed by a single application of the Exterminator. I place a high value upon your remedy, and recommend it as the best article I know of as a vermin destroyer.

"Yours truly, B. R. BARLOW, *Superintendent.*"

With this we dismiss the subject, with the remark that the liquid is most purely vegetable, contains not an atom of mineral, mercury, oil, or opium; an infant might be bathed in it without any injury whatever; besides, it cleanses whatever it is applied to; is the most universally used as a hair tonic and scalp-cleaner, as to its chief article, than any other hair-wash in existence. The strong presumption is that it is as good for destroying the various vermin which infest beasts as well as men. It instantly kills and keeps away bed-bugs as well as the genus louse. It has been officially tried on the horses in the army; with like effect as to the heads of children.

Since writing the above, Mr. Dodge assures us that it is the greatest blessing in the world for

Poodle dogs,
Pet birds, and
Tom-cats, as it rids them of fleas in an extraordinarily short space of time; and that henceforward ladies may kiss and fondle them, and carry them on their bosoms with impunity, having the assurance that they will not be flead, since "Dodge's Infallible Vermin Exterminator" is in the house.

Doubtless time will prove that it is efficacious for the destruction of all vermin and insects, whether they infest domestic animals or In fact, there is evidence to believe that the Exterminator is

A UNIVERSAL BOON.

It will doubtless be found in all the drug-stores in the nation, at twenty-five cents a bottle, which contains enough for five heads. In the language of Burns, will be efficacious in places where, heretofore,

"Nor horn nor bane
Ne'er dared unsettle
Their thick plantations."

☞ Sold, wholesale and retail, at 831 Broadway, New-York.

---

THE PATENT POCKET STEREOSCOPE

is the novelty already referred to, which, although it is not pecuniarily valuable, is pleasurably so, because it is really a

STEREOPTICON IN EVERY HOUSE.

It gives you as much the real appearance of any place stereoptically or photographically taken, as if it were the reality looked upon by the actual living eye. Such at least it appears to us to do; with the incalculable advantage that the pictures can be looked at *just as fast as any one desires to pick them up from the table* and hold them before the eye; no other adjustment is needed; hence it is a source of the most agreeable and pleasurable amusement, and is as inexhaustible as the supply of stereoscopic pictures; and these already are for sale in our shops, having been taken in all parts of the world of note enough to be visited by travelers. In short, a man can see all the grand sights of the world at the expense of one dollar or over, according to quality and the price of the pictures, and yet remain in his own parlor; he can look at them over and over again with unwearying satisfaction, the greater if he has ever seen them once, because he recognizes them as old friends, and they thus bring back delightful reminiscences of the

"Days of auld lang syne."

### Friends of Soldiers.

On a late march one thousand soldiers were sun-struck; one hundred died. Green leaves in the hat would have prevented all. The son of a New-York capitalist generously gave his shelter to a sick comrade, and remained in the rain all night, with his feet in the water, sickened and died. "Soldier Health," twenty-five cents, by the editor of Hall's Journal of Health, shows how the soldier may avoid sickness; how act in emergencies; to staunch a wound with a stick, if left alone; to arrest a common army disease with a bit of cloth; to heal a cut with a little powder; and a hundred other expedients, which, if read over attentively, could be carried in the head, and applied from memory. No man ought to go into the army without reading and impressing its contents on the mind.

### Particular Notice.

Subscribers will please understand that the size of the Journal from the first has been twenty-four pages of reading matter. We have not allowed advertisements to encroach on that space; on the contrary, by setting up our Health Tracts in small type, we have given each year from ten to twenty more pages of reading matter than we engaged to do, and all this without increasing the price of our Journal as almost all publishers have done. In this way our subscribers can not lose by the advertisements we give, while they may be largely benefited pecuniarily.

